# Mucopolysaccharidosis-Plus Syndrome

**DOI:** 10.3390/ijms21020421

**Published:** 2020-01-09

**Authors:** Filipp Vasilev, Aitalina Sukhomyasova, Takanobu Otomo

**Affiliations:** 1 Department of Molecular and Genetic Medicine, Kawasaki Medical School, Kurashiki, Okayama 701-0192, Japan; vasilyevmd@gmail.com; 2 International Research Fellow of Japan Society for the Promotion of Science (Postdoctoral Fellowships for Research in Japan (Standard)), Tokyo 102-0083, Japan; 3 Laboratory of Genome Medicine, North-Eastern Federal University, 677013 Yakutsk, Sakha Republic, Russia; aitalinas@yandex.ru

**Keywords:** mucopolysaccharidosis-plus syndrome, MPSPS, VPS33A, glycosaminoglycans, HOPS, lysosomal storage disorders

## Abstract

Previously, we reported a novel disease of impaired glycosaminoglycans (GAGs) metabolism without deficiency of known lysosomal enzymes—mucopolysaccharidosis-plus syndrome (MPSPS). MPSPS, whose pathophysiology is not elucidated, is an autosomal recessive multisystem disorder caused by a specific mutation p.R498W in the *VPS33A* gene. VPS33A functions in endocytic and autophagic pathways, but p.R498W mutation did not affect both of these pathways in the patient’s skin fibroblast. Nineteen patients with MPSPS have been identified: seventeen patients were found among the Yakut population (Russia) and two patients from Turkey. Clinical features of MPSPS patients are similar to conventional mucopolysaccharidoses (MPS). In addition to typical symptoms for conventional MPS, MPSPS patients developed other features such as congenital heart defects, renal and hematopoietic disorders. Diagnosis generally requires evidence of clinical picture similar to MPS and molecular genetic testing. Disease is very severe, prognosis is unfavorable and most of patients died at age of 10–20 months. Currently there is no specific therapy for this disease and clinical management is limited to supportive and symptomatic treatment.

## 1. Introduction

Lysosomal storage disorders (LSD) are a group of inherited metabolic disorders that are mainly caused by enzyme deficiencies within the lysosome resulting in accumulation of undegraded substrate [1]. Mucopolysaccharidoses (MPS) are rare LSD caused by a deficiency of specific lysosomal enzymes that affect degradation of glycosaminoglycans (GAGs). GAGs are long heteropolysaccharides composed of repeating disaccharide monomers found in connective and other tissues. These GAGs include dermatan sulfate, heparan sulfate, keratan sulfate, chondroitin sulfate, and hyaluronic acid. The accumulation of GAGs in various organs and tissues of patients leads to a broad spectrum of clinical manifestations. Onset of MPS symptoms mostly occurs in children aged 2–4 years and then progressing. Diagnosis is typically based on clinical examination, urine tests and the enzymatic activity in blood cells or fibroblasts. Eleven enzyme deficiencies that cause seven distinct clinical types of MPS have been identified (MPS I (subtypes: Hurler, Hurler-Scheie, Scheie); MPS II; MPS III (subtypes: IIIA, IIIB, IIIC, IIID); MPS IV (subtypes IVA, IVB); MPS VI; MPS VII; MPS IX). All these MPS disorders are inherited in an autosomal recessive manner with the exception of MPS II, which is X-linked [2].

In early 2010s children with severe disease leading to death before the age of two years LSD phenotype was established in Republic of Sakha (Yakutia, Russia). They were diagnosed as “undifferentiated inherited disease of metabolism”.

In 2017, after describing the clinical picture and identifying the cause of the disease, a new disease was included in the Online Mendelian Inheritance in Man^®^ (OMIM^®^) database as entry #617303. The new disease was described as mucopolysaccharidosis-plus syndrome (MPSPS). The name of disease, MPSPS means that in addition to typical symptoms for conventional MPS, patients developed other features such as congenital heart defects, renal and hematopoietic disorders.

The symptoms associated with MPSPS were first described in 2014 by Dr. Gurinova [3]. They described clinical phenotype of eleven patients (four males, seven females) with Hurler-like phenotype. All eleven patients with MPSPS were found among native population of Yakutia (Northeast Russia). Eight children died before the age of eighteen-month. All children were born in Yakut families from young parents. Congenital hypothyroidism, cystic fibrosis, alpha-1-antitrypsin deficiency, and chromosomal pathology were excluded by laboratory testing. Cetylpyridinium chloride (CPC)-citrate turbidity test were used to detect increase of GAGs in urine of two patients. Anamnestic and clinical data of these children made it possible to suspect LSD with a Hurler-like phenotype.

In 2017, we reported two new children with novel disease and summarized all patients from Russia with clinical features of conventional MPS, impaired GAG metabolism and a lack of all known lysosomal enzyme deficiencies [4]. Dursun et al. [5] reported two sisters, born of consanguineous Turkish parents, with an autosomal recessive inborn error of metabolism resulting in a multisystem disorder with features of the MPS or LSD. We and Dursun et al. identified by using whole-exome sequencing that novel autosomal recessive multisystem disorder caused by a specific mutation p.R498W in the vacuolar protein sorting-associated protein 33A (*VPS33A*) gene.

## 2. Disease Overview

### 2.1. Etiology

**Figure 1:**
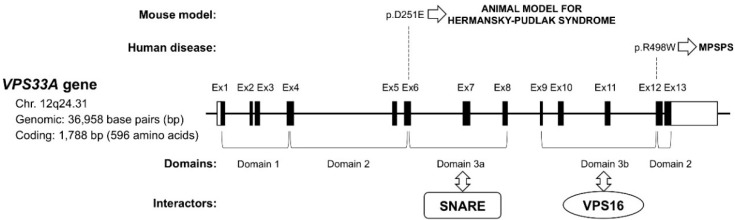
Schematic representation of the *VPS33A* gene. Exons (Ex, black boxes) and untranslated regions (white boxes) are indicated. Horizontal brackets indicate structural domains of the VPS33A protein, and known interaction partners are shown below. The p.D251E and p.R498W mutations are responsible for animal model (buff mouse) for Hermansky–Pudlak syndrome and human mucopolysaccharidosis-plus syndrome (MPSPS), respectively.

MPSPS is inherited in an autosomal recessive manner. MPSPS is caused by homozygous specific mutation in the gene encoding the VPS33A (NM_022916.4: c.1492C>T, NP_075067.2: p.Arg498Trp, hereinafter referred to p.R498W). To date, only p.R498W has been described as a mutation that leads to the development of this disease. The gene that codifies VPS33A protein is located on chromosome 12q24.31 and contains 13 exons. The mutation is located in exon 12, which encodes domain 2 of the VPS33A protein (Figure 1).

### 2.2. Epidemiology

MPSPS is a very rare disease. Currently, nineteen patients with MPSPS have been identified. Seventeen patients were found among the Yakut population (Russia) and two patients from Turkey [4,5]. The detection of the disease among these populations may be explained by the fact that the Yakuts and Turks belong to the Turkic ethnic group. Incidence rate of MPSPS in Yakuts population is predicted as 1 per 12,100 birth. We previously analyzed 110 healthy Yakuts for this mutation and revealed an allele frequency of 1 in 110 [4]. We then analyzed a larger Yakut population (202 Yakut individuals) by using real-time polymerase chain reaction (PCR) and identified extremely high allele frequency in this population (1:81).

The Yakuts are representatives of the Central Asian anthropological type of the North Asian race and live in the northeastern part of Siberia in the Republic of Sakha of the Russian Federation. Yakut population is approximately 480,000 people. The issue of Yakut ethnogenesis is still unclear and controversial. The most traditional theory considers the Yakuts as a people who once lived in the Baikal region and gradually migrated to northern Siberia in the 13th or 14th century Anno Domini (AD). Studies of the Y chromosome revealed that the population went through the so-called bottleneck effect [6]. The Yakut population is characterized by a high degree of genetic homogeneity of the population due to geographical isolation and low migration. These factors also determined the effect of the founder and the very high frequency of hereditary diseases [7,8,9].

### 2.3. Pathophysiology

#### 2.3.1. HOPS and CORVET

VPS33A is a member of the Sec1/Munc18-related (SM) protein family and a core component of the class C core vacuole/endosome tethering (CORVET) and the homotypic fusion and protein sorting (HOPS) complexes [10,11]. The CORVET and HOPS complexes are large, multi-protein machines that are capable of interacting with multiple other factors of the endolysosomal pathway. Both complexes are heterohexamers and share four subunits. VPS33A, VPS11, VPS16 and VPS18 interact with Rab GTPase interaction module through VPS3/VPS8 and VPS39/VPS41 for CORVET and HOPS, respectively [12,13]. CORVET is a Rab5 effector complex and functions in early endosomes [14,15]. HOPS can bind efficiently to late endosomes and lysosomes through Rab7 [13,16,17]. Thus, main function of CORVET and HOPS is homotypic and heterotypic fusions of early endosomes and late endosomes/lysosomes, respectively. The HOPS complex also plays an important role in phagosomal biogenesis through transition from Rab5 to Rab7 endosomes [18,19]. HOPS and CORVET subunits are required for early embryonic development and their knock-out is usually lethal in mice [20,21]. CORVET/HOPS subunits have a similar structure: a β-propeller domain at the N terminus with zinc-finger domain at the C-terminal [22,23]. Electron microscopy analyses revealed that HOPS and CORVET shares a similar architecture and forms an elongated particle with two Rab-binding sites formed by Vps39/Vps41 (HOPS) and Vps3/VPS8 (CORVET) at opposite ends [24,25].

Phenotype caused by mutations in the genes of other members of these complexes is different from phenotype of MPSPS patients. Patients with homozygous missense mutation in VPS16 show adolescent-onset primary dystonia [26]. In the research of Peng et al. [27] knock-out of Vps18 in mice leads to severe neurodegeneration and neuronal migration defects. Several researchers reported patients with mutations in *VPS11* show autosomal-recessive leukoencephalopathy, hypomyelination and developmental delay [28,29,30]. It was also found that patients with missense mutations in VPS8 had multiple joint contractures, delayed motor development, craniofacial dysmorphism and cerebellar vermis hypoplasia [31]. Vps3 and Vps8 regulate vesicular transport and their depletion inhibit transport of integrins from early to recycling endosomes [32]. Pols et al. [33] revealed that VPS41 is essential for transfer of lysosomal membrane proteins from trans-Golgi network to lysosomes. It was shown that role of Vps3/Vps8 as well as Vps41 in trafficking is independent of CORVET and HOPS. Accumulation of GAGs was not investigated in all of these pathologies. Homozygous carriers of p.(Leu387_Gly395del) in the *VPS11* gene had elevated urinary levels of glycosphingolipids as compared to an age-matched control [30].

#### 2.3.2. VPS33A Protein and Function

Among vacuolar protein sorting 33 proteins, Vps33p was first described in *Saccharomyces cerevisiae* [34]. In yeast was found only one form of Vps33p [35]. It was shown that yeast Vps33p functions in vacuolar biogenesis and Golgi-to-vacuole protein transport [36]. Pevsner et al. [37] cloned first mammalian Vps33a, a homolog of yeast Vps33p. In metazoans, VPS33A has two homologs: VPS33A and VPS33B. Rat VPS33a shows 31% identity and 50% similarity to the human VPS33B [38]. VPS33A and VPS33B share 30% identity [10]. VPS33A is required for Rab-driven membrane-tethering and fusion that is mediated by CORVET and HOPS is of central importance for the biogenesis of endosomes and lysosomes [39]. VPS33A also interacts with syntaxin 17 (STX17) and promotes soluble N-ethylmaleimide-sensitive fusion protein attachment protein receptors (SNARE)-mediated autophagosome-lysosome fusion [16].

Homozygous spontaneous mutation p.D251E in *Vps33a* used as an animal model (buff mouse) for Hermansky–Pudlak syndrome [40,41]. Hermansky–Pudlak syndrome is characterized by oculocutaneous albinism, with concomitant visual impairment and excessive bleeding [42]. Buff mice also exhibits striking behavioral deficiencies and Purkinje cell degeneration [43]. Missense mutation in *Vps33A* homolog cause carnation eye color in *Drosophila* [44].

Mutations in *VPS33B* cause ARC [45] and ARKID syndromes [46]. ARC (arthrogryposis-renal dysfunction-cholestasis) syndrome characterized by renal tubular dysfunction, cholestasis, and platelet storage pool deficiency. ARKID (autosomal-recessive keratoderma-ichthyosis-deafness) syndrome is a severe disorder with phenotype including ichthyosis, hearing loss, platelet dysfunction and osteopenia.

VPS33A protein consists of 596 amino acids and has a molecular mass of 67.6 kDa. The *VPS33A* gene encodes the VPS33A protein composed of four domains: 1, 2, 3a and 3b [47,48]. Different domains within the VPS33A protein are responsible for its different functions. Domain 3a of VPS33A is predicted to be the interacting interface to the SNARE complex [16,47]. Domain 3b of VPS33A is the binding interface to VPS16 (subunit of HOPS complex) [48]. The mutation of *VPS33A* gene detected in MPSPS patients is located in domain 2 which is thought not to interact with VPS16 and SNARE complex (Figure 1). Currently domain 1 and domain 2 of VPS33A are domains of unknown function.

#### 2.3.3. MPSPS Investigations

In our study was shown that p.R498W mutation affects GAG metabolism [4]. Arginine at position 498 is highly conserved among many species. Mutation p.R498W was predicted to be disease-causing and reducing protein stability by analyzing with several in silico tools. MPSPS patients showed excess secretion of urinary GAG and extremely high levels of plasma heparan sulfate. These results suggested that dysfunction of VPS33A protein leads to GAG accumulation and causes MPS-like phenotypes. However, substrates accumulation was not caused by decrease of lysosomal glycosidase activities involved in GAG degradation. Localization of lysosomal enzymes, cathepsin D processing and lipid trafficking were not affected. p.R498W mutation also did not affect endocytic and autophagic pathways. Investigation of the autophagic pathway revealed normal autophagic degradation in patient-derived skin fibroblasts. Investigation of binding mutant VPS33A with known interactors, VPS16 (HOPS, CORVET) and STX17, showed that these interactions were not altered. Slight lysosomal over-acidification was observed in patient-derived and VPS33A-depleted cells.

Pavlova et al. [49] showed that patient-derived fibroblasts have increased vacuolization and abnormal endocytic trafficking of lactosylceramide. Quantity of enlarged vacuoles (>500 nm diameter/cell) was increased in patients cells. It was also noted accumulation of cholesterol in MPSPS patients. They revealed pathological excess of sialylated conjugates, dermatan and heparan sulfates in the urine. Lysosomal enzymes activities were within normal ranges. Mass spectrometry analysis showed high levels of unacylated lyso-glycosphingolipid, *β*-d-galactosylsphingosine in skin fibroblasts of MPSPS patients. The glucosylceramide/ceramide ratio was not affected in cells from patients. The 3D crystal structure analysis showed that replacement of arginine 498 by tryptophan is predicted to disrupt the salt bridge with aspartate at position 484 and hydrogen bond network and thus de-stabilizes VPS33A folding. Level of VPS33A protein was reduced in patients but level of VPS33A mRNA did not differ from controls. Also reduced level of VPS18 (HOPS, CORVET) and VPS41 (HOPS) proteins were observed in skin fibroblast of MPSPS patients. It was suggested that reduced level of VPS33A protein comes from increased proteasomal degradation of mutant VPS33A and this leads to instability of the HOPS/CORVET complexes.

It is still unknown how missense mutation p.R498W in VPS33A cause accumulation of GAGs. Detailed mechanism and disease pathophysiology remain to be elucidated.

### 2.4. Clinical Picture

**Figure 2:**
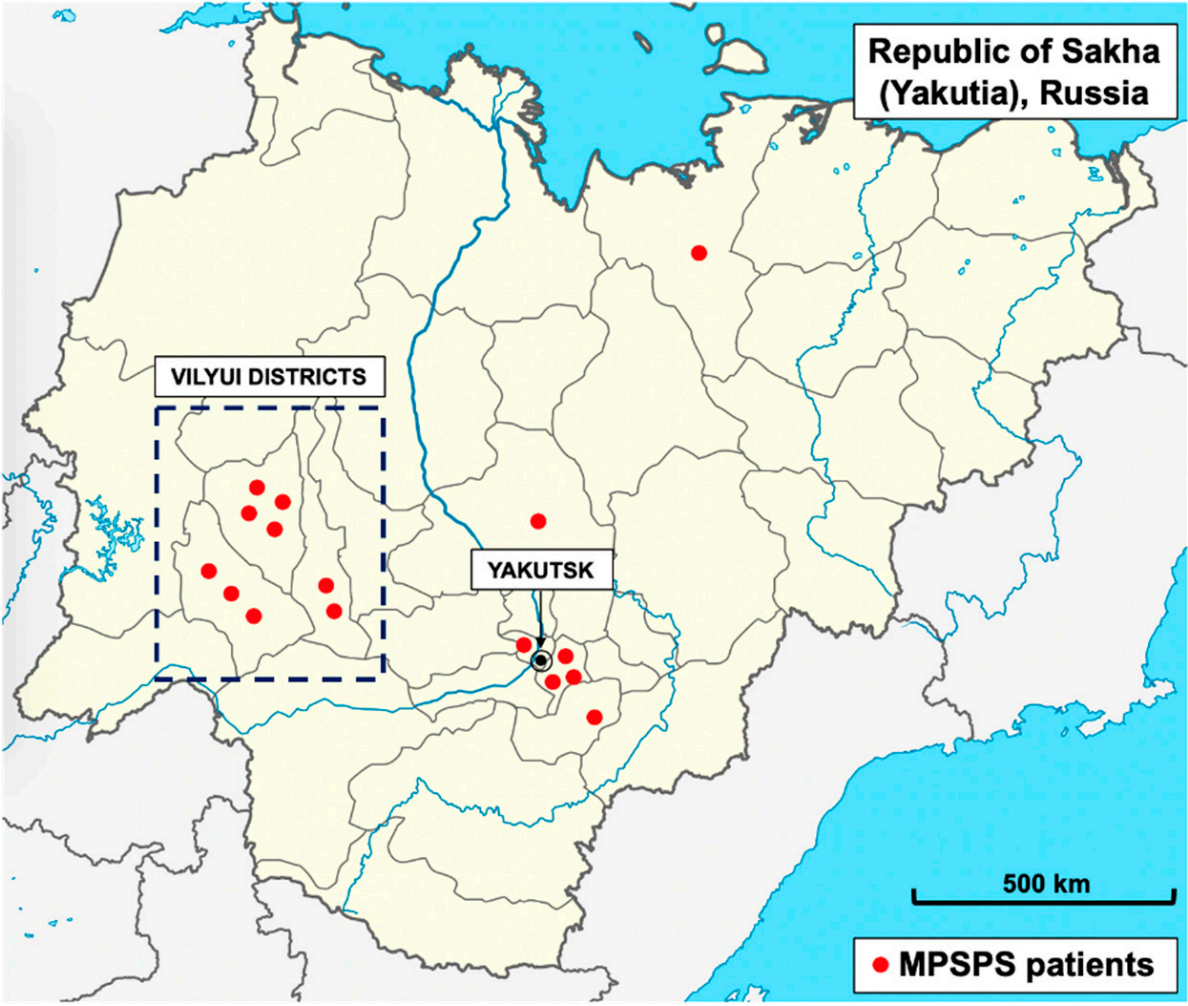
Distribution of MPSPS patients in Yakutia. Red rounds indicate MPSPS patients. Most patients were found in the Vilyui group of districts (blue dotted rectangle) and near city of Yakutsk (circled dot), the capital of Yakutia (North-Eastern Siberia, Russia).

In the Medical Genetic Center in Yakutsk (Russia) were counseling sixteen patients with MPSPS disease: eight girls and eight boys. All the children were born to non-consanguineous healthy parents of Yakut ethnicity. Genealogical history of intermarriage was denied, but in most cases, both parents were natives from one district of Yakutia (Figure 2).

Half of the children born from first pregnancy. During pregnancy birth defects have not been identified by ultrasound investigation. MPSPS patients were born healthy at term. Apgar scores were within the normal range (≥7).

**Figure 3:**
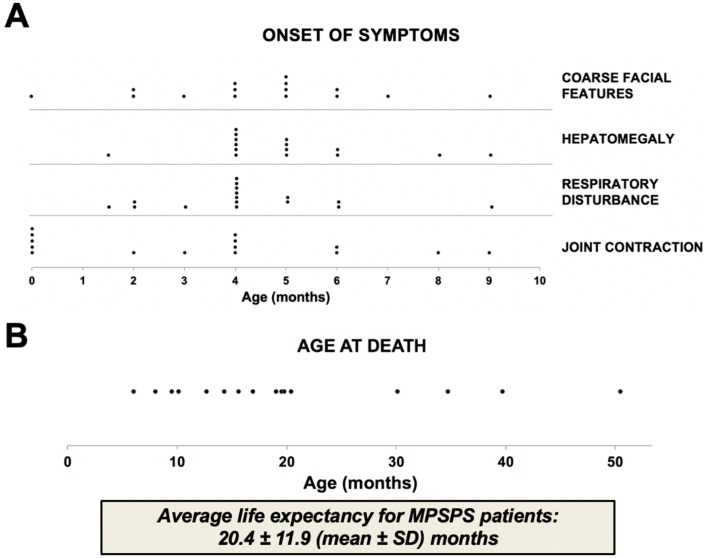
Onset of clinical features and life expectancy of MPSPS patients. Data from sixteen MPSPS patients from Yakutia (Russia). (**A**) Symptoms of disease usually occur between the ages of 2–6 months. (**B**) Most of patients died of cardiorespiratory failure at age of 10–20 months. Each dot indicates one MPSPS patient.

Patients came for genetic counseling or admitted to hospital cause frequent recurrent respiratory diseases at the age of 2–6 months (Figure 3A). Usually they had a complaint of frequent cough, shortness of breath, noisy breathing and stiffness of joints.

Such acute conditions since the beginning tended to more frequent and each time the child’s condition worsened. Most patients developed congenital heart defects. Mitral and tricuspid valve defects and pulmonary hypertension progressed from moderate to severe within 3–4 months.

**Figure 4:**
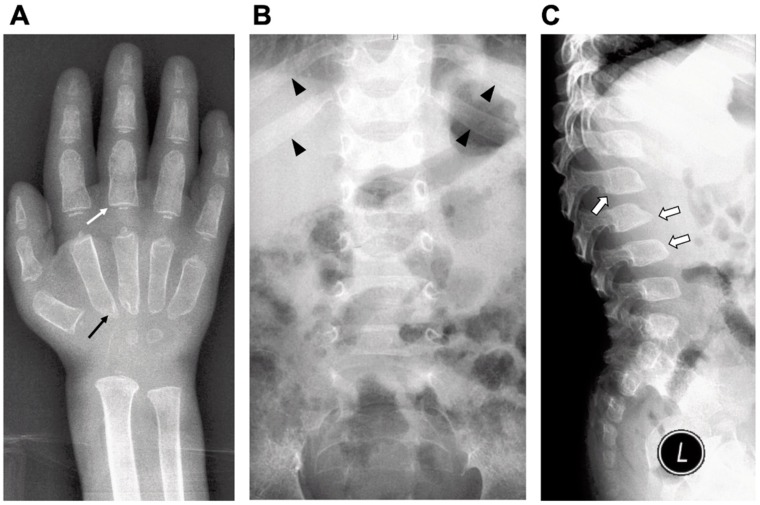
Radiographic findings of a MPSPS patient at age of one-year and nine-month. Characteristics of dysostosis multiplex: (**A**) bullet-shaped phalanges (white arrow) and the metacarpal pointing (black arrow); (**B**) widening of anterior ribs (oar-shaped ribs) (arrowhead) and (**C**) vertebral dysplasia with round- and hooked-shape vertebral bodies (open arrow).

All MPSPS patients had a clinical phenotype similar to MPS patients:

- Coarse facial features: short nose and neck, prominent forehead, epicanthal folds, telecanthus, thick hair, excessive hair growth, periorbital puffiness, long eyelashes, large rounded cheeks, full lips, macroglossia
- Skin: thick
- Skeletal: barrel-shaped chest, pectus carinatum, kyphosis (thoracic, lumbar), lordosis (lumbar), multiple dysostosis (Figure 4), joints contracture, stiffness of the elbow, wrist, hip, knee and ankle joints, finger phalangeal swelling, limitation of motion of fingers, claw-hand deformities, deep palmar furrows
- Hernia: may be present (inguinal hernia was reported in 2 out of 17 patients’ medical records)
- Respiratory system: bronchial obstruction, noisy breathing, shortness of breath, dry and moist rales
- Cardiovascular system: tachycardia, hypertension, systolic murmur, cardiomegaly (ventricular hypertrophy), valve insufficiency (tricuspid, mitral), congenital heart defects (patent ductus arteriosus (PDA) and atrial septal defect (ASD), pulmonary hypertension
- Neurology: hydrocephalus syndrome, hypotonia, poor tendon reflexes, nystagmus
- Central nervous system: psychomotor retardation, developmental delay

Dursun et al. [5] reported two Turkish female siblings with clinical and radiological findings similar to phenotype of MPSPS patients from Yakutia. Patients were born from consanguineous marriage partners (cousins) at term by Cesarean section. Pregnancies were uneventful. Patients suffered from respiratory difficulties accompanied by pulmonary infections. On examinations the elder patient had a wide range of clinical presentations: coarse face, short neck, large forehead, a broad nasal bridge, long eyelashes, synophrys, hirsutism, thick hair, pectus excavatum, clubbing and long fingers, macroglossia, mild hepatosplenomegaly, limitation of movement of the knees and hips and mild talipes equinovarus. The younger sister had signs of multiple dysostosis, laryngomalasia and bifid epiglottis. These siblings also had multisystem involvement including respiratory, renal, central nervous and hematopoietic systems.

### 2.5. Case Report

**Figure 5:**
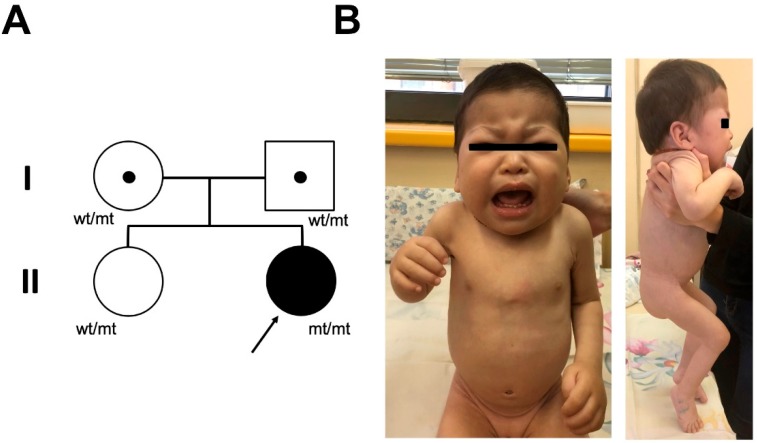
Pedigree of the family and clinical phenotype of the patient. (**A**) Pedigree of the probands family. wt, wild-type allele; mt, mutant allele, c.1492C>T, p.R498W in *VPS33A*. (**B**) Clinical phenotype of patient at age of one-year nine-month. Coarse facial features, periorbital puffiness, thick skin, short neck, deformation of the chest wall, contractures at the fingers, elbow and knee. Informed consent with permission to publish patient photos and case details was obtained from parents.

Here we presented a new case of MPSPS with a typical presentation of the clinical features. The proband, a five-month-old girl, was admitted to neurology department of hospital in Yakutia because of hypertonia of legs. High muscle tone was noticed by parents from birth. A girl was born healthy at term with a birth weight of 3970 g. Apgar scores of 8 and 8 at 1 and 5 min, respectively. Toxicosis was observed in the I trimester of pregnancy. The girl is a second child of healthy Yakut non-consanguineous parents. She has an older healthy sister. There was no family history of diseases similar to MPSPS (Figure 5A).

Clinical data was collected twice from the mother of patient, medical history and physical examinations, and summarized in Table 1.

Physical examination at five-month showed height 68 cm (+1.8 SD) and weight 7200 g (+0.4 SD). Standard deviation scores were calculated using World Health Organization (WHO) growth standards. The physical appearance characterized mild coarse facial features, periorbital puffiness, pale and thick skin, thick cheeks, short neck, mild pectus carinatum, lumbar kyphosis, joint stiffness, contractures at the fingers, elbow and knee and deep palmar creases (Figure 5B). The abdomen was slightly enlarged. Respiratory system examination revealed noisy breathing and shortness of breath without pathological breath sounds. Medical history showed that she did not suffer from frequent respiratory infections but had a shortness of breath from three months. From twelve months of age she began to be repeatedly hospitalized because of respiratory tract infections.

Computed tomography (CT) of the lungs revealed atelectasis S1–S2 of right lung. X-ray tests of the spine showed wedge-shaped deformity of the body of L2 and L3 vertebras. Echocardiography revealed patent foramen ovale (PFO). Heart cavities were not dilated, Ejection fraction (EF) 72%. Electrocardiography examination was normal. Abdominal ultrasound showed moderate hepatomegaly. Electromyoneurography and neurosonography within normal parameters. Her routine laboratory studies showed anemia (Hb 9.9 g/dL), eosinophilia (14%), hypoalbuminemia (37 g/L) and elevated AST (131.3 U/L). Other parameters were within normal limits. Urine analysis was normal.

Condition of the child was getting worse. At age of one-year and nine-month the body measures were all distinctly below of normal values: height 79 cm (−1.5 SD) and weight 9000 g (−1.4 SD). She had a severe developmental delay: cannot stand, sits up with support and delayed speech. Abdominal ultrasound revealed hepatosplenomegaly, nephromegaly and renal ectasia. Echocardiography displayed coronary artery fistula (0.19–0.22 сm), insufficiency of aortic valve, mitral and tricuspid valve regurgitation, pulmonary hypertension, EF 77%.

On the basis of clinical phenotype, probable diagnosis of MPSPS was made. Genomic DNA from peripheral blood of patient and parents was analyzed by real-time PCR and direct DNA sequencing. DNA analysis showed that patient and parents are carriers of homozygous and heterozygous p.R498W mutation of *VPS33A* gene, respectively. Qualitative and quantitative analysis of GAGs in urine and enzymes activities were not tested. Patient developed respiratory insufficiency with infection followed by multiple organ failure and died at the age of one year and ten months.

## 3. Diagnosis

Diagnosis generally requires evidence of clinical picture and molecular genetic testing. The finding of elevated urinary GAGs is supportive. Diagnosis is based upon on identification of detailed patient and family history. Prenatal diagnosis can verify if a fetus is affected with MPSPS.

### 3.1. Diagnosis and Clinical Features

All MPSPS patients showed recurrent infections of the upper respiratory tract in infancy or early childhood. MPSPS is a multisystem disorder with severe phenotype. In addition to clinical phenotype of conventional MPS, MPSPS patients present with hematopoietic disorders, renal involvement and congenital heart defects. Individuals affected by MPSPS share similar symptoms that progress rapidly. A detailed description of the clinical picture of MPSPS patients is presented in Section 2.4.

### 3.2. Laboratory and Instrumental Examinations

Most patients develop hematopoietic disorders including anemia, thrombocytopenia and leukocytopenia. An increase in serum IgM and decrease of IgG levels were found in MPSPS patients [49]. Bone marrow aspiration analysis showed hypoplastic bone marrow without any storage cells. Radiograph examinations showed signs of dysostosis multiplex: J-shaped sella turcica, bullet-shaped phalanges and the metacarpal pointing, metaphyseal widening of the long bones with cortical thinning, oar-shaped ribs, flared iliac wings and malalignment of vertebrae and vertebral dysplasia with round- and hooked-shape vertebral bodies (Figure 4). Magnetic resonance imaging (MRI) and CT of the brain showed apparent delayed myelination in the peripheral white matter and calcification in the basal ganglia. Echocardiography usually identifies atrial septal defect, patent ductus arteriosus or hypertrophic cardiomyopathy. Retinal hypopigmentation was seen on the fundus photograph [4]. Electron microscopy of conjunctiva biopsy revealed early and late endosomes in endothelial cells, myelin figures and enlarged mitochondria. The majority of MPSPS patients had a nephrotic syndrome. Proteinuria were in the range from mild to severe. Patients have an increased excretion of uronic acid in urine. Renal ultrasonography shows nephromegaly and increased echogenity [4]. Renal biopsy showed segmental sclerosis, periglomerular fibrosis and inflammatory cell infiltration.

### 3.3. GAGs and Enzymes Activity

Examination of urine can reveal elevated levels of GAGs, specifically heparan and dermatan sulfate [4,5,49]. Accumulation of heparan sulfate was also detected in patient’s plasma and skin fibroblasts. Moreover, the heparan sulfate level in plasma was significantly exceeded in comparison with conventional MPS [4]. Activities of lysosomal glycosidases involved in the degradation of GAGs are not decreased in plasma, lymphocytes and patient-derived fibroblasts [4,5]. Finding of MPS-like phenotype and excess secretion of urinary GAGs without deficiency in the activity of lysosomal enzymes is the basis for suspicion of MPSPS. However, these tests cannot be used to provide definitive diagnosis of MPSPS. Diagnosis should be confirmed by molecular DNA analysis.

### 3.4. Genetic Testing

MPSPS is caused by missense mutation p.R498W in the *VPS33A* gene and is inherited in an autosomal recessive pattern. This mutation is specific, and no other disease-causative mutation has been reported in the *VPS33A* gene. Parents of MPSPS patients are heterozygous carriers of p.R498W mutation. Heterozygous carriers for MPSPS mutation are generally disease free and do not develop symptoms of disease. MPSPS affects males and females in equal numbers. The frequency of the pathogenic allele was very low in diverse populations from different databases (Table 2). But among Yakut population we observed extremely high allele frequency.

### 3.5. Differential Diagnosis and Prognosis

Clinical features of MPSPS patients are similar to conventional MPS. In addition to other forms of MPS, the differential diagnosis should also include LSD, such as mucolipidosis types II and III, Niemann-Pick disease, Gaucher’s disease, fucosidosis, mannosidosis, sialidosis and multiple sulfatase deficiency. Clinical and biochemical studies distinguish these conditions. When disease is very severe, prognosis is unfavorable. Most of patients died of cardiorespiratory failure at the age of 10–20 months (Figure 1).

## 4. Treatment and Management

Currently there is no established any biomarkers and specific therapy for this disease. All patients should be monitored with assessment for disease progression. As a result of the multisystemic involvement of MPSPS, treatment and care with multidisciplinary care teams is recommended. Clinical management is limited to supportive and symptomatic treatment. Treatment involves bronchial drainage, oxygen therapy, antibiotics for respiratory infections, administration of vitamins and angiotensin-converting-enzyme (ACE) inhibitors. A blood transfusion may be needed to treat hematologic disorders. Patients with congenital heart defects may require surgical treatment.

Pathophysiology of the disease is still unknown, however possible treatments of MPSPS are being investigated. In experimental study of Pavlova et al. [49] was shown that treatment of MPSPS patient-derived fibroblasts with proteasomal inhibitor (bortezomib), and inhibitor of glucosylceramide synthesis (eliglustat) partially corrected the impaired lactosylceramide trafficking defect.

Since a mutation that causes disease has been identified, prenatal diagnosis is now available. A detailed family history should be obtained, and genetic counseling should be offered to couples with heterozygous p.R498W mutations. Psychological support is essential for parents and family members of MPSPS patient. Implementation of screening programs to populations considered at risk of MPSPS can increase early detection and intervention.

## Figures and Tables

**Table 1:** Clinical characteristics of the proband.

Symptoms	Age of Proband at Time of Examination	
	Five-Month	One-Year Nine-Month
Recurrent respiratory infections	Absent	Present
Coarse facial features	Mild	Present
Dysostosis multiplex	Present	Present ^1^
Joints contraction	Present	Present
Hepatomegaly	Present	Present
Heart defects	PFO ^2^	Insufficiency of valves ^3^
Anemia	Mild	Present
Thrombocytopenia	Absent	Absent
Leukocytopenia	Absent	Absent
Nephromegaly	Absent	Present
Nephritic syndrome	Absent	Present
Proteinuria	Absent	Present
Psychomotor delay	Absent	Present

^1^ indicated in Figure 4; ^2^ PFO, patent foramen ovale; ^3^ aortic, mitral and tricuspid insufficiency.

**Table 2:** Allele frequency of c.1492C>T *VPS33A* (p.R498W, rs767748011).

Population (Database)	Allele Count	Allele Numbers	Allele Frequency
ExAC database	2	118,008	1:59,004
TOPMED database	1	125,568	1:125,568
GnomAD database	2	245,570	1:122,785
Ensembl database			
African	0	15,260	0:15,260
European (Finnish)	0	22,218	0:22,218
European (Non-Finnish)	2	111,244	1:55,622
Ashkenazi Jewish	0	9828	0:9828
East Asian	0	17,236	0:17,236
South Asian	0	30,770	0:30,770
Latino	0	33,544	0:33,544
Yakut (our study)	5	404	1:81

## Abbreviations

ACE	Angiotensin-converting-enzyme
AD	Anno Domini
AST	Aspartate aminotransferase
CT	Computed tomography
CORVET	Class C core vacuole/endosome tethering
DNA	Deoxyribonucleic acid
EF	Ejection fraction
GAG	Glycosaminoglycans
HOPS	Homotypic fusion and vacuole protein sorting
LSD	Lysosomal storage disorder
MPS	Mucopolysaccharidosis
MPSPS	Mucopolysaccharidosis-plus syndrome
MRI	Magnetic resonance imaging
PFO	Patent foramen ovale
PCR	Polymerase chain reaction
SNARE	Soluble *N*-ethylmaleimide-sensitive fusion protein attachment protein receptors
VPS33A	Vacuolar protein sorting-associated protein 33A
WHO	World Health Organization
